# Probable *Bartonella clarridgeiae* Prosthetic Valve Endocarditis and Aortic Root Abscess, Australia, 2020

**DOI:** 10.3201/eid3205.251558

**Published:** 2026-05

**Authors:** Mark Cribb, Sarah Coghill

**Affiliations:** Lismore Base Hospital, Northern New South Wales Health District, Lismore, New South Wales, Australia

**Keywords:** Bartonella clarridgeiae, endocarditis, bacteria, zoonoses, aneurysm, RNA, ribosomal, 16S, Bartonella, Australia

## Abstract

We describe a case of endocarditis and aortic root abscess caused by *Bartonella clarridgeiae* bacteria in a patient in Australia. The patient initially sought care for leg pain and was found to have bilateral tibioperoneal trunk mycotic aneurysms. 16S rRNA PCR on excised aneurysm tissue identified the cause as *B. clarridgeiae*.

*Bartonella* species are gram-negative, fastidious, facultative intracellular bacteria (*1*). They are a cause of blood culture–negative endocarditis, infective endocarditis with negative blood cultures resulting from antibiotic exposure or fastidious pathogens (*2*). *B. clarridgeiae* is an emerging pathogen in the genus, first identified in 1995 and attributed as a human pathogen in 1997 (*3*,*4*). Tibioperoneal trunk (TPT) aneurysms are a rare clinical phenomenon; causes include trauma, vasculitis, and infective endocarditis or mycotic aneurysm (*5*). We describe a case of bilateral TPT aneurysms, prosthetic valve infective endocarditis, and aortic root abscess in a patient in Australia in 2020 that was caused by *B. clarridgeiae*, identified on 16S ribosomal RNA of aneurysm tissue samples.

A man in his 80s sought care at a local emergency department for pain in his left calf for 1 month. He had aortic stenosis requiring a transcatheter aortic valve implantation 2 years earlier. He had sought care several times over the previous 8 months with calf pain, chest pain, and lethargy. On examination, he was noted to have a swollen and tender left calf, a pansystolic murmur, 2 splinter hemorrhages, and a temperature of 37.9°C. He initially received intravenous cefazolin for possible cellulitis, later changed to amoxicillin/clavulanate. 

Computed tomography (CT) angiogram demonstrated a hematoma in the left calf associated with a TPT pseudoaneurysm and a right TPT thrombus. The left TPT aneurysm was surgically repaired. Transesophageal echocardiography (TOE) showed no valvular incompetence or vegetations. 

CT positron emission tomography scan demonstrated focal intense fluorodeoxyglucose activity at the transcatheter aortic valve implantation (SUVmax = 5.9) (Figure, panel A), postsurgical changes involving the left lower leg (Figure, panel B), and focal moderate activity at the right tibioperoneal trunk, indicating another mycotic aneurysm. We reviewed TOE findings again and identified an echolucent space at the sinus of Valsalva, consistent with an aortic root abscess. Therapy was switched to intravenous ceftriaxone and vancomycin. The patient was deemed not to be a cardiac surgery candidate.

**Figure:**
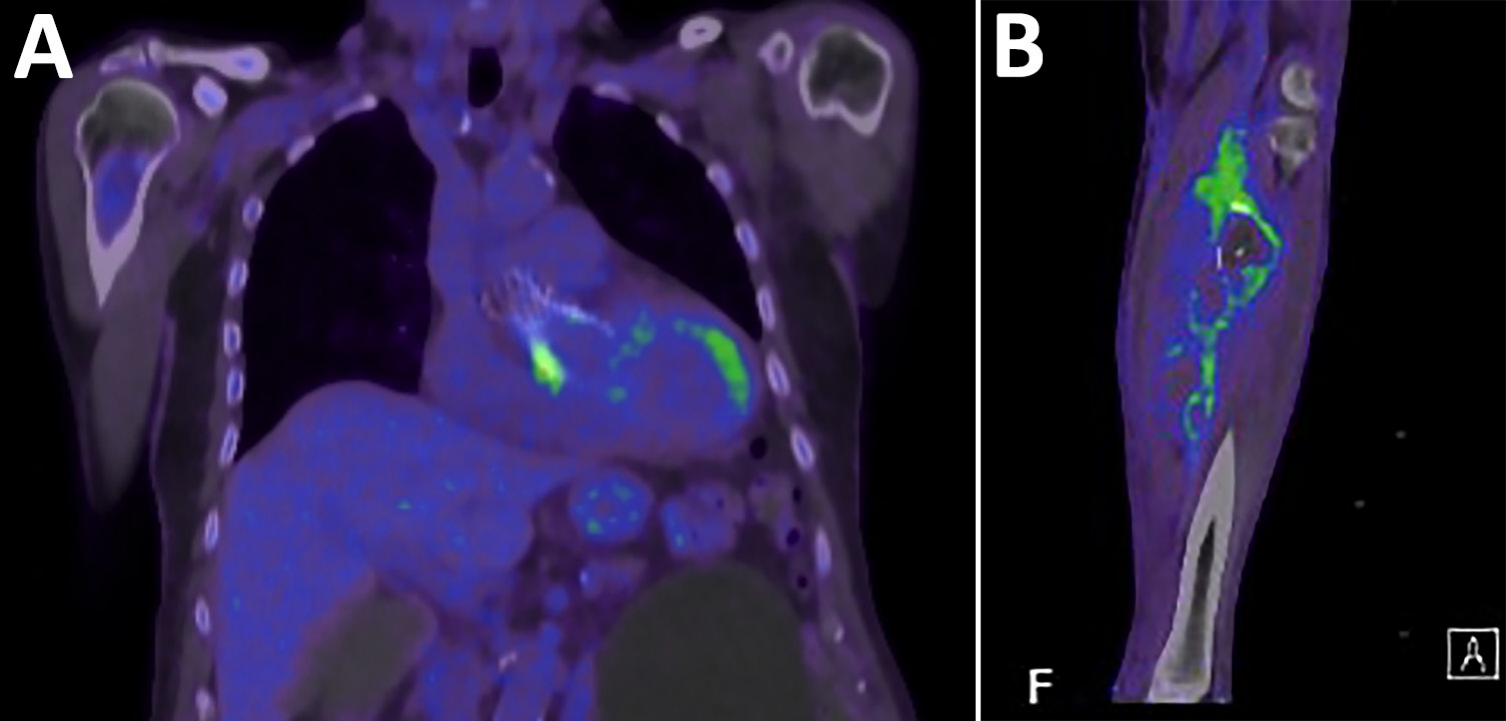
Positron emission tomography/computed tomography images from study of patient with *Bartonella clarridgeiae* prosthetic-valve endocarditis and aortic root abscess, Australia, 2020. A) Fluorodeoxyglucose activity at ventricular end of the transcatheter aortic valve implantation; B) repaired tibioperoneal trunk aneurysm in patient’s left leg.

Four sets of blood cultures were negative for bacteremia. Operative tissue culture tested negative for bacterial growth using standard media; we sent the tissue samples for 16S rRNA PCR. We conducted serologic testing for *Brucella* spp., *Coxiella burnettii*, syphilis, and HIV. We tested for *Bartonella* spp. using FOCUS Diagnostics Indirect Immunofluorescence Assay IgG kit for *B. henslae* and *B. quintana* IgG (http://focusdiagnostics.in). 16S rRNA PCR detected *B. clarridgeiae* DNA in operative tissue samples. Serology results for *B. henslae* bacteria were strongly positive (IgG >1:2,048 [<128]); all other serology results, including *B. quintana* testing, were negative. We performed PCR testing of blood with primers and probe targeting a conserved portion of the citrate synthase gene; results for *Bartonella* spp. DNA were negative. 

We switched treatment to intravenous gentamicin with oral doxycycline (100 mg 2×/d). The patient experienced ongoing fevers and elevated C-reactive protein levels. Because of concern for treatment failure, we added oral ciprofloxacin for 6 weeks; repeated TOE showed stable changes. After 6 weeks, the patient continued oral doxycycline (100 mg 2×/d) for suppression; he remained well at a 2-year follow-up visit.

Early reports for bartonellosis associated *B. clarridgeiae* with cat-scratch disease (*6*). Cats are a reservoir for *B. clarridgeiae* and *B. henslae* (*4*,*7*); the patient we report kept multiple cats at home, although he recalled no preceding cat bite or scratch. A case of endocarditis and aortic root abscess caused by *B. clarridgeiae* diagnosed in 2019 was treated successfully with ceftriaxone, doxycycline, and heart valve replacement (*7*). 

Serology has traditionally been a method for diagnosing bartonellosis; however, cross-reactivity can occur (*8*), as in this case. Molecular diagnostics are a valuable tool in accurate diagnosis of *Bartonella* endocarditis; emergence of pathogens such as *B. clarridgeiae* may be related to their increasing use (*1*,*8*). A limitation of our report is that the average nucleotide identity percentage is unavailable to confirm distinction between *Bartonella* species. 

At the time of this case in 2020, the recommended treatment for *Bartonella* endocarditis was gentamicin for 2 weeks with doxycycline for 6 weeks (*3*). Because *Bartonella* endocarditis has been associated with infection-related glomerulonephritis (*9*), newer recommendations suggest doxycycline or azithromycin for 12 weeks and rifampin for 6 weeks (*2*). Other suggested therapies for *Bartonella* infections have included trimethoprim/sulfamethoxazole and ciprofloxacin (*3*). Because of concerns for treatment failure in our patient, we added ciprofloxacin empirically, without strong evidence available to guide treatment.

Delay in diagnosis of endocarditis is an unfortunate theme in mycotic aneurysm with low-medium virulence organisms (*10*). Our patient had a protracted manifestation over months, with nonspecific symptoms before diagnosis. This case reinforces the need for suspicion of endocarditis in patients seeking care for TPT aneurysms and highlights the pathogenicity of *B. clarridgeiae* bacteria in this context. Suspicion of and investigation for causes of blood culture–negative endocarditis including *Bartonella* spp. is therefore warranted in patients with TPT aneurysms, should initial microbiologic investigations be negative. Molecular diagnostics including 16S rRNA PCR can aid diagnosis.
